# High Power Density X-Band GaN-on-Si HEMTs with 10.2 W/mm Used by Low Parasitic Gold-Free Ohmic Contact

**DOI:** 10.3390/mi16091067

**Published:** 2025-09-22

**Authors:** Jiale Du, Hao Lu, Bin Hou, Ling Yang, Meng Zhang, Mei Wu, Kaiwen Chen, Tianqi Pan, Yifan Chen, Hailin Liu, Qingyuan Chang, Xiaohua Ma, Yue Hao

**Affiliations:** School of Microelectronics, Xidian University, Xi’an 710071, China

**Keywords:** GaN-on-Si, gold-free, regrown ohmic contact, RF loss, output power density (*P*_out_)

## Abstract

To enhance the RF power properties of CMOS-compatible gold-free GaN devices, this work introduces a kind of GaN-on-Si HEMT with a low parasitic regrown ohmic contact technology. Attributed to the highly doped n^+^ InGaN regrown layer and smooth morphology of gold-free ohmic stacks, the lowest ohmic contact resistance (*R*_c_) was presented as 0.072 Ω·mm. More importantly, low RF loss and low total dislocation density (TDD) of the Si-based GaN epitaxy were achieved by a designed two-step-graded (TSG) transition structure for the use of scaling-down devices in high-frequency applications. Finally, the fabricated GaN HEMTs on the Si substrate presented a maximum drain current (*I*_drain_) of 1206 mA/mm, a peak transconductance (*G*_m_) of 391 mS/mm, and a breakdown voltage (*V*_BR_) of 169 V. The outstanding material and DC performances strongly encourage a maximum output power density (*P*_out_) of 10.2 W/mm at 8 GHz and drain voltage (*V*_drain_) of 50 V in active pulse mode, which, to our best knowledge, updates the highest power level for gold-free GaN devices on Si substrates. The power results reflect the reliable potential of low parasitic regrown ohmic contact technology for future large-scale CMOS-integrated circuits in RF applications.

## 1. Introduction

Gallium nitride (GaN) HEMTs on Si substrates have appealed to be an effective technology for mm-wave, high power, and large-scale heterogeneous integration circuit applications due to their large wafer scalability, cost-effectiveness, promising RF power properties, and natural CMOS-compatible substrate platform [1,2]. However, most reported Si-based GaN devices are based on the gold-containing ohmic contact which is incompatible with CMOS circuits and, more than that, formed by a high-temperature alloying process with a high thermal budget, rough morphology, and high-frequency parasitic effect [3,4]. Thus, a gold-free ohmic contact technology, along with low ohmic contact resistance (*R*_c_) and low thermal budget, is crucial for the realization of GaN-on-Si RF devices with high output power and efficiency.

Available low ohmic contact methods mainly include ion implantation, barrier-recessed ohmic contact, and regrown highly doped n^+^ GaN. However, for GaN-on-Si devices, ion implantation for highly doped AlGaN in the ohmic region is hard to realize. This is because the activation process requires a high temperature (>1200 °C), which exceeds the typical growth temperature of Si-based GaN, thereby degrading the quality of the epitaxy material [4]. Barrier-recessed ohmic technologies were applied with different gold-free metal stacks for GaN-on-Si devices with low *R*_c_ [5], but etch-induced damage and high thermal budget remain to be solved. Among these techniques, regrown n^+^ GaN ohmic contact is acknowledged as an effective method for low *R*_c_ and smooth morphology, avoiding an additional high-temperature alloying process [6,7]. However, reports are quite limited on regrown ohmic contact with gold-free metal stacks for the GaN-on-Si platform.

In this work, a gold-free regrown ohmic contact technology was implemented for GaN HEMTs on a Si substrate. Owing to the highly doped n^+^ InGaN layer and Ti/Al/Pt/Ti ohmic metal stack, a lowest *R*_c_ of 0.072 Ω·mm was obtained without an additional high-temperature annealing process, which effectively suppresses the high-frequency parasitic effect. Except for the low *R*_c_, crystalline quality and RF loss from Si-based GaN epitaxy materials remain critical to mitigate the high-frequency parasitic effect, especially for device scaling design in GaN/Si-CMOS integration applications. A high-quality ultrathin buffer technology using a two-step-graded (TSG) transition structure was introduced to reduce the dislocation density of the GaN-on-Si epitaxy platform, which has been proposed in our previous work [8]. Based on the TSG transition technology, a remarkably low total dislocation density (TDD) of 1.68 × 10^9^ cm^−2^ was achieved, which compares favorably with other state-of-the-art GaN-on-Si RF devices. To further investigate the parasitic effect of Si-based GaN epitaxy material, RF loss was evaluated by coplanar waveguide (CPW) transmission line structures on the GaN epitaxy on the Si substrate with a low value of 0.24 dB/mm at 8 GHz. The advanced crystalline quality and low RF loss manifest the advancement of TSG transition technology. Consequently, the fabricated gold-free AlGaN/GaN HEMTs exhibited a saturated drain current (*I*_drain_) of 1206 mA/mm and breakdown voltage (*V*_BR_) of 169 V, resulting in a maximum power density (*P*_out_) of 10.2 W/mm with peak PAE of 50.5% at 8 GHz, which are the highest reported values among gold-free GaN-on-Si devices.

## 2. Material Growth and Device Fabrication

**Figure 1:**
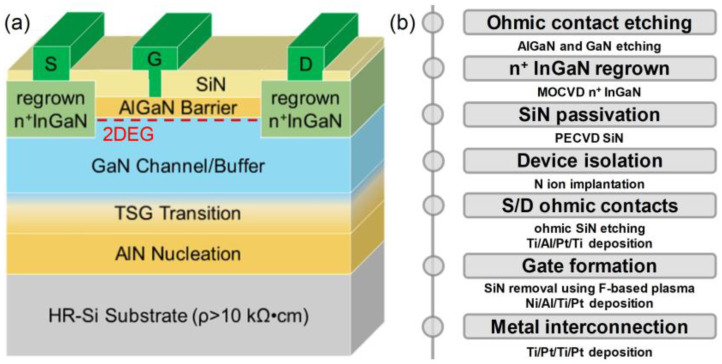
(**a**) Schematic and (**b**) process flow of the gold-free AlGaN/GaN HEMTs on Si substrate using regrown ohmic contact technology with dimensions of *L*_g_ = 0.25 μm, *W*_g_ = 100 μm, and *L*_sd_ = 4 μm.

As described in Figure 1a, the AlGaN/GaN epitaxy structure was grown on a high-resistivity (HR) Si substrate (*ρ* > 10 kΩ·cm) through metal oxide chemical vapor deposition (MOCVD). The epitaxial structure comprises an AlN nucleation layer, the TSG transition layers, a GaN buffer layer, an unintentionally doped (UID) GaN channel layer, and an AlGaN barrier layer from bottom to top. To suppress dislocation density induced from large lattice mismatch between GaN and the Si substrate, the TSG transition structure was applied to acquire a high-quality epitaxy [8]. The detailed TSG buffer includes the 30 nm Al_0.4_Ga_0.6_N stress layer and an AlN/Al_0.2_Ga_0.8_N composed layer of 90 nm total. The TDD of GaN epitaxy structures was calculated with a low value of 1.68 × 10^9^ cm^−2^ in ω scanning mode [9], proving the high growth quality of TSG transition technology. Hall measurements under room temperature presented the sheet electron concentration and electron mobility of 1.14 × 10^13^ cm^−2^ and 1950 cm^2^/V·s, respectively.

Figure 1b exhibits the fabrication process flow of the gold-free GaN devices with the regrown ohmic contact in detail. The regrown process flow began with the removal of the AlGaN barrier layer and GaN channel layer in the ohmic region by photolithography and Cl-based etching. To ensure better contact between the regrown n^+^ InGaN and the 2DEG of the channel, the low-damage etching condition was developed. The n^+^ InGaN with Si doping concentration of 2 × 10^20^ cm^−3^ was grown at 800 °C by MOCVD. After that, the two-step wet rinse processes were implemented for removal of regrown n^+^ InGaN outside the ohmic contact regions. Prior to regrowth, a SiO_2_ mask layer was deposited to protect the access region. In the first step, hydrofluoric acid (HF) was used to selectively remove the undesired regrown material covering the access region, leveraging its vigorous reaction with SiO_2_. Subsequently, a mixture of acetone and isopropyl alcohol was applied to thoroughly cleanse the area and eliminate any residual reactants or contaminants.

To protect the surface of the AlGaN barrier layer, a SiN passivation layer was deposited by plasma-enhanced chemical vapor deposition (PECVD). Prior to the SiN passivation, in situ NH_3_ plasma pre-treatment was introduced in the chamber to improve the passivation effect, and device planar isolation of the active region was carried out via nitrogen ion implantation. Then, the Ti/Al/Pt/Ti gold-free ohmic metal stacks of source and drain pads were deposited on the regrown n^+^ InGaN. For gate fabrication, the gate foot definition was realized by electron beam lithography (EBL), and then the SiN of this region was removed by F-based plasma. And the T-gate structure was fabricated by the deposition of Ni/Al/Ti/Pt gold-free metal stacks to ensure the complete CMOS-compatible flow scheme. Finally, the interconnection of the metal was accomplished as Ti/Pt/Ti/Pt by electron beam evaporation to achieve a low parasitic resistance. The devices feature the gate length (*L*_g_), gate width (*W*_g_), and source–drain spacing (*L*_sd_) of 0.25 μm, 100 μm, and 4 μm, respectively.

## 3. Results and Discussion

**Figure 2:**
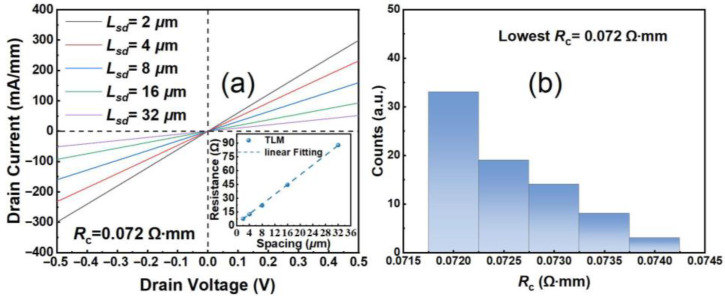
(**a**) TLM I-V measurements of the Ti/Al/Pt/Ti ohmic contact resistivity, and (**b**) a distribution of the *R*_c_ for the gold-free GaN HEMTs on Si substrate.

**Figure 3:**
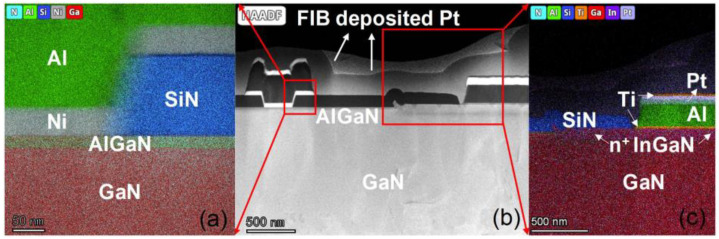
(**a**) EDS element mapping micrograph of the gate foot region, (**b**) the HAADF-STEM image of the whole region consisting of the gate and ohmic region, and (**c**) EDS micrograph of the ohmic region for the fabricated GaN-on-Si devices.

Figure 2a demonstrates the I-V characteristics of Ti/Al/Pt/Ti ohmic contact resistivity as a function of increasing spacings from 2 μm to 32 μm, measured by the transmission line model (TLM). Profiting from the highly doped regrown n^+^ InGaN and low-resistive ohmic metal stack, the extracted lowest *R*_c_ was 0.072 Ω·mm, with high uniformity across the whole wafer as presented in Figure 2b. The specific contact resistivity of the regrown ohmic contact is also evaluated from the TLM structures as 1.87 × 10^−7^ Ω·cm^−2^. The high-angle annular dark-field scanning transmission electron microscope (HAADF-STEM, Talos F200X G4 TEM, Thermo Fisher Company) and the energy dispersive X-ray spectroscopy (EDS) element mapping of the regrown n^+^ InGaN and ohmic metal stack are exhibited in Figure 3b,c.

**Figure 4:**
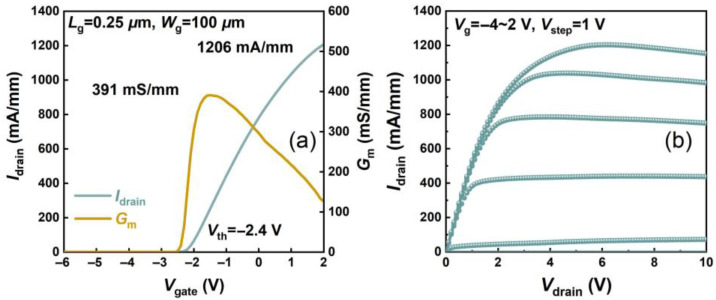
(**a**) Linear transfer I-V and (**b**) output I-V properties of the gold-free GaN HEMTs on Si substrate.

**Figure 5:**
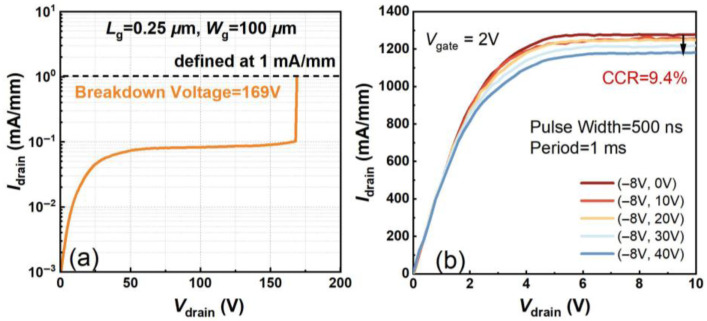
(**a**) Off-state breakdown properties and (**b**) double-pulsed output I-V characteristics of gold-free GaN HEMTs on Si substrate with *L*_sd_ = 4 μm.

The gold-free T-gate microstructure was also depicted by HAADF-STEM as shown in Figure 3a,b, demonstrating uniform coverage of the Ni/Al/Ti/Pt gate metal. The DC I-V characteristics of the device were assessed by the Keysight B1500A semiconductor parameter analyzer (Keysight Technologies (China) Co., Ltd.). Figure 4a shows transfer I-V properties of the gold-free GaN-on-Si devices under *V*_drain_ = 6 V and gate-to-source voltage sweep from −6 V to 2 V. The threshold voltage (*V*th) was extracted as −2.4 V. It can be seen that the *I*_drain_ and the *G*_m_ of the device were measured as 1206 mA/mm and 391 mS/mm, respectively, which demonstrates the excellent electrostatic control of the gold-free gate electrode to the 2DEG channel. Off-state breakdown characteristics of the fabricated GaN HEMT with *L*_sd_ = 4 μm were measured under *V*_gate_ = −8 V. Leveraging the TSG transition structure’s high-quality GaN epitaxy, we achieved *V*_BR_ = 169 V defined at 1 mA/mm, confirming excellent high-voltage performance for RF power applications (Figure 5a). To assess the dynamic current collapse of the gold-free GaN devices, double-pulsed output I-V characteristics were measured by the Keithley 4200-SCS system, with a pulse width of 500 ns and a duty cycle of 0.05%. Due to the low-dislocation-density GaN epitaxy and high-quality SiN passivation, the small current collapse ratio (CCR) of *V*_gate_ = 2 V of merely 9.4% was achieved up to the quiescent bias (*V*_GQ_ = −8 V, *V*_DQ_ = 40 V), predicting high dynamic operation for RF domains.

**Figure 6:**
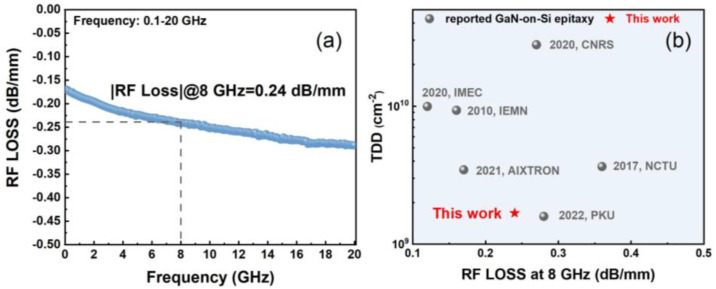
(**a**) RF loss versus frequency from 0.1 to 20 GHz; (**b**) benchmark of TDD and RF loss at 8 GHz among reported GaN-on-Si epitaxial materials [10,11,12,13,14,15].

The CPW transmission line model was adopted to measure RF loss at different frequencies for the grown GaN-on-Si epitaxy materials. Based on S-parameter measurements using Agilent E8363B PNA network analyzer (Agilent Technologies, Inc.) calibrated with the through–reflect–line (TRL) calibration standard, the RF loss was measured from 0.1 GHz to 20 GHz. A low value of RF Loss at 8 GHz was 0.24 dB/mm, as marked in Figure 6a. As shown in Figure 6b, the RF loss at 8 GHz and the low total dislocation density of the GaN buffer in this work both stay at a high level among reported epitaxial structures for GaN-on-Si RF applications [10,11,12,13,14,15]. The results demonstrate that the TSG structure on the HR-Si substrate achieves both low RF loss and high crystalline quality, facilitating high RF power performance.

**Figure 7:**
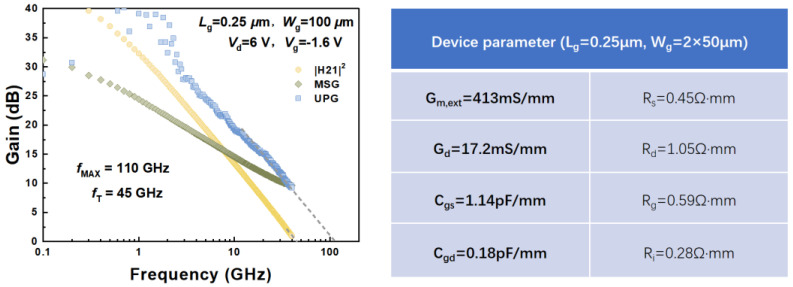
Measured *f*_T_/*f*_max_ of the gold-free GaN HEMTs on Si substrates at *V*_DS_ = 6 V after de-embedding. The right table demonstrates the extracted intrinsic parameters.

The small-signal performance of the fabricated gold-free GaN HEMTs on silicon substrates has been thoroughly characterized. RF measurements of the GaN-on-Si HEMT were conducted from 0.1 to 40 GHz using an Agilent 8363B network analyzer with short–open–through calibration. The measured data were de-embedded using cold and hot FET techniques [16]. As shown in Figure 7, the small-signal RF performance of the 0.25 μm devices is presented, with S-parameters measured from 0.1 to 40 GHz at *V*_DS_ of 6 V. The resulting *f*_T_ and *f*_max_ values are 45 GHz and 110 GHz, respectively. The extracted intrinsic parameters were summarized in the right table of Figure 7.

**Figure 8:**
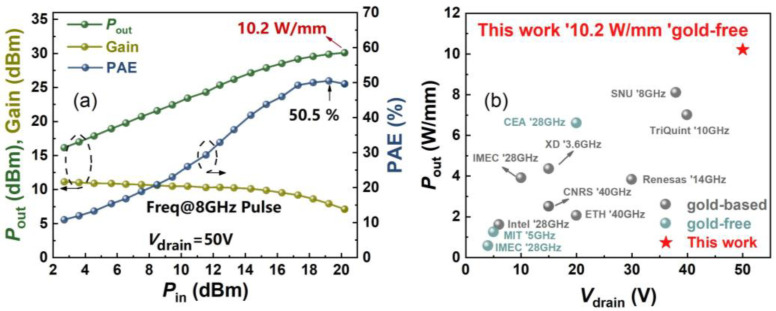
(**a**) Large-signal power measurements at 8 GHz of the gold-free GaN-on-Si devices by active load-pull system in pulse mode. (**b**) Benchmark of *P*_out_ versus *V*_drain_ among state-of-the-art GaN-on-Si reports [5,17,18,19,20,21,22,23,24,25,26].

Figure 8a exhibits the large-signal power performance of the gold-free AlGaN/GaN devices at 8 GHz measured by a Maury active load-pull system in pulse mode. The pulse width and duty cycle are 20 μs and 10%, respectively. Ascribing to the low contact resistance, high breakdown voltage, and low RF loss, the device exhibited a high *P*_out_ of 10.2 W/mm and a peak PAE of 50.5% at the operation voltage of 50 V, as demonstrated in Figure 8a. As indicated in Figure 8b, the gold-free GaN devices in this work present the highest output power density among the reported RF GaN-on-Si devices [5,17,18,19,20,21,22,23,24,25,26]. Such a high-power performance verifies the advancement of the low parasitic regrown ohmic contact technology in gold-free GaN devices for GaN/Si-CMOS heterogeneous integration applications. With the high demand for exploring higher frequency, building upon these advanced technologies, the combination of high-quality GaN-on-Si epitaxial growth and ultra-low contact resistance provides a solid foundation for future high-frequency applications.

## 4. Conclusions

In this paper, a GaN-on-Si HEMT employing low parasitic regrown ohmic contact technology and a TSG transition structure was presented. The highly doped regrown n^+^ InGaN layer and Ti/Al/Pt/Ti ohmic metal structure contribute to the low *R*_c_ of 0.072 Ω·mm. In addition, the high-quality GaN layer and low RF loss ensure high *I*_drain_ and *V*_BR_ for high-voltage RF operation. Eventually, the proposed gold-free GaN devices on Si substrates achieve a highest power performance of 10.2 W/mm with peak PAE of 50.5% at 8 GHz and 50 V operation. These results break the performance limitations of gold-free GaN-on-Si RF devices, demonstrating the potential of low-resistive regrown ohmic contact technology for future heterogeneous integration applications.

## Data Availability

Data are contained within the article.
